# The Wicked Problem of Selection and Competency-Based Education: Applying TRIZ Inversion to the Graduate Medical Education Selection Process

**DOI:** 10.5334/pme.2596

**Published:** 2026-09-29

**Authors:** Holly A. Caretta-Weyer, Ming-Ka Chan, Mary Ellen J. Goldhamer, Alan Schwartz, Teresa M. Chan, Lalena M. Yarris, Ian W. Incoll, Arvin Damodaran, H. Carrie Chen, As members of the International Competency-Based Health Professions Educators Collaborative

**Affiliations:** 1 Stanford University School of Medicine, Palo Alto, California, USA; 2 Max Rady College of Medicine, Rady Faculty of Health Sciences, University of Manitoba, Canada; 3 Harvard Kidney Urology Hematology Training Institute, Boston, Massachusetts, USA; 4 Departments of Medical Education and Pediatrics, University of Illinois Chicago, Chicago, Illinois, USA; 5 Toronto Metropolitan University, Brampton, ON, Canada; 6 Department of Medicine, Division of Emergency Medicine/Division of Education and Innovation, McMaster University centre for Education Research, Innovation, and Theory (MERIT), Faculty of Health Sciences, McMaster University, Hamilton, ON, Canada; 7 Oregon Health and Science University, Portland, Oregon, USA; 8 Faculty of Health and Medicine, University of Newcastle, Australia; 9 Graduate Programs in Surgical Education, University of Melbourne, Australia; 10 School of Clinical Medicine, Faculty of Medicine and Health, UNSW Sydney, Australia; 11 Kaiser Permanente Bernard J. Tyson School of Medicine, Pasadena, California, USA

## Abstract

**Introduction:**

Graduate medical education (GME) selection processes may pose significant barriers to implementing competency-based health professions education (CBHPE). Current selection metrics are largely nonpredictive of future success, and the removal of clerkship grades has given rise to a shadow economy of effort focused on publications, research hours, and other activities that detract from clinical training. This misalignment between selection practices and CBHPE principles constitutes a wicked problem that requires innovative approaches to develop new solutions.

**Methods:**

We conducted a TRIZ (Theory of Inventive Problem Solving) exercise, a Liberating Structure that challenges assumptions by asking what would make a process fail spectacularly. Eight participants representing three countries, two continents, and four medical specialties convened via video conference in October 2025. Participants served as proxies for key stakeholder groups (students, program directors, medical school leadership, and patients/society), identified elements of the worst-possible selection system, compared them to current practices, and proposed solutions.

**Results:**

Participants identified numerous elements characterizing suboptimal selection systems, including inequitable and biased processes, arbitrary decision-making, gaming of metrics, resource-intensive processes that fail to differentiate candidates, misinformation from training sites, and prioritization of self-interest over patient needs. Many of these elements were recognized as present in current GME selection systems worldwide.

**Discussion:**

Recommendations to align selection with CBHPE included embedding social accountability throughout selection, leveraging coproduced competency frameworks, prioritizing workforce diversity, ensuring equity in assessment and selection, incentivizing a growth mindset through a transparent learner handover, and implementing structural innovations, including preference signaling or potentially lottery systems, to better align CBHPE and selection.

## Introduction

Graduate medical education (GME) is undergoing a sea change in its delivery with the evolution of competency-based health professions education (CHPBE); however, innovations in GME trainee selection have not kept pace [1]. The process for trainee selection in GME could present a barrier to the implementation of CBHPE as it may create perverse incentives that are misaligned with the societal needs that CBHPE is intended to serve [2]. For example, the literature shows repeatedly that the current metrics used to select candidates for GME positions are largely nonpredictive of future success [3,4,5,6]. Moreover, as medical training programs worldwide have begun implementing CBHPE, new challenges in selection have arisen. The removal of clerkship grades, intended to facilitate a growth orientation and mitigate bias, has instead given rise to a shadow economy of effort in pursuit of a GME training position [7]. This shadow economy places a greater emphasis on research hours; publications; letters of recommendation from known entities in the field; and other activities in the service, policy, and leadership spaces [7,8]. All of this focus takes attention away from students’ clinical training and subsequently impacts their preparedness for patient care expectations in GME training.

Although we agree that CBHPE is necessary to prepare medical school graduates for the next phase of training, we must also create and leverage GME selection systems in which students thrive and can provide safe and effective patient care. To reinvigorate analysis of this ‘wicked problem’, we conducted a TRIZ exercise within a diverse group of medical educators to broaden our thinking.

## Methods

### Theoretical Approach

Wicked problems cannot be solved using traditional methods. They are different from traditional problems in that they are: 1) defined differently from multiple perspectives; 2) appear differently in each different context, but follow consistent patterns wherever they appear; and 3) can never be completely solved [9,10]. The issue of selection in medical education, particularly in light of the adoption of CBHPE, falls clearly into this category. While the literature on wicked problems is more nuanced, we provide this abbreviated working definition for this manuscript to frame our thinking as it evolved over several meetings discussed below. Given that the problem cannot be completely solved, the temptation is to give up hope and stop trying altogether. To combat that, we chose an alternative approach.

One such method, which has been leveraged across numerous disciplines to move past an impasse toward solutions, is Liberating Structures [11]. Liberating Structures are activities that can be used to enable people, regardless of their background or roles, to work together in finding creative solutions that they can then adapt to local contexts and utilize to move forward. The premise is that individuals can brainstorm new ideas, coalesce around the most attractive or viable options, leverage other individuals or processes needed to make progress, and clearly delineate what they absolutely should *not* do moving forward. Next steps are then identified, successful practices are adopted, and ideally momentum shifts.

There are 33 different types of Liberating Structures. One of these is known as TRIZ, or the Theory of Inventive Problem Solving [12]. In the TRIZ exercise, innovators ask one central question: *What would we need to do to make a process fail spectacularly?* [12]. This approach provides innovators with the chance to “invert” the main problem and exposes assumptions in a way that traditional brainstorming cannot, thus revealing new areas where innovation and intervention may, ironically, change things for the better. A TRIZ addresses key activities and behaviors that limit success in a given organization, domain, or process using a nonthreatening approach to challenge what have previously been viewed as untouchable assumptions and practices. The idea is to push participants to creatively dismantle the protected practices to move forward.

### ICBHPE Summit

The International Competency-Based Health Professions Education (ICBHPE) Collaborative convened a summit in February 2025 at Stanford University with invited attendees representing five countries. At this meeting, a working group comprising eleven individuals met during a breakout session and discussed perspectives on selection and CBHPE. We agreed that multiple challenges in this area are shared globally and discussed ways we might become “unstuck” in our thinking to move past them and advance improvements. We decided to complete a TRIZ exercise asynchronously after the summit.

### The TRIZ Exercise

We reached out to the entire ICBHPE Collaborative listserv (90 members from six continents) via email in early October 2025 and subsequently convened eight voluntary, self-selected participants on October 30, 2025, via Zoom video conference to conduct the TRIZ exercise. Participants represented three countries on two continents and four medical specialties. All participants are current faculty at medical schools. Using the TRIZ approach, we asked participants to serve as proxies for key interest-holder groups and to create a list of all practices in selection to achieve the worst possible CBHPE outcome through the lens of key interest-holders (e.g., students/applicants, GME program directors, medical school leadership/deans, and patients/society). TRIZ participants were prompted to think about the selection process in its entirety and also consider each granular component separately, including assessment practices, materials provided in the selection process, what is considered or prioritized in the selection process, application screening, interviewing, ranking processes, bias in assessment and selection, workforce issues, outcomes at any stage of the processes, and anything else that came to mind. Once this list was created, we asked participants to review it and identify what is currently occurring within our systems and/or processes that may resemble the listed items. Finally, we asked TRIZ participants to explore ways to help mitigate these undesirable practices and move toward more productive solutions with the goal of incremental change in the selection process.

### Data Collection

The 60-minute TRIZ exercise was recorded and transcribed verbatim. The transcripts were compared to real-time field notes taken by the lead author (HCW) simultaneously during the exercise to ensure completeness and accuracy. The exercise continued through each phase from the perspective of each different interest holder until all ideas were exhausted, mirroring a sense of real-time thematic sufficiency [13].

### Analysis

A number of core themes were distilled using an inductive approach by the primary author (HCW) across interest-holder groups for in-depth exploration and consolidated by a subsequent author (HCC). However, the broader dataset is presented in Appendix 1. The primary author (HCW) grouped generated topics into related domains with multiple items in each. Subsequently, the senior author (HCC) consolidated the items under each larger domain into key themes for reporting purposes. All other authors reviewed this data for clarity and accuracy. Member checking was performed with all participants as the TRIZ data were analyzed to ensure accurate representation of the discussion [14]. This was done by providing the larger domains and consolidated themes to the group for their review and any revisions based on their understanding of the discussions and topic generation during the TRIZ. In the following text, we provide a focused review of the discussion of the core components of the maladaptive, or most suboptimal, system, how this resembles our current selection processes, and what we can do about it.

### Reflexivity

All of the authors are currently faculty within medical schools in the United States, Canada, or Australia. Each role; the author serves as a medical education researcher with a focus on competency-based health professions education. HCW, TMC, AD, and HCC all serve in leadership roles within a dean’s office or equivalent at their medical schools undergraduate medical education programs and learners. MKC, TMC, MEG, LMY, and IWI all serve in leadership and oversight roles for graduate medical education programs at their institutions. AS oversees the education of graduate students in education. Several of the authors hold formal or informal roles within their specialty societies or national regulatory bodies. While not all interest-holder perspectives are directly represented, each author currently serves in a role listed below or works directly with the population represented.

### Ethics

The present work was reviewed by the institutional review board at Stanford University and deemed exempt.

## Results

We identified several key elements of the worst-possible selection system from the perspective of numerous stakeholders, including students, GME program directors, undergraduate deans, and patients. All data extracted from the TRIZ can be found in Appendix 1.

### Students’ Perspective of the Worst Selection System or Process

From students’ perspective, the theme of unfairness was prevalent across all proposed elements. The selection process being clearly inequitable and biased, or entirely arbitrary, was set forth as an element of a suboptimal selection process. Systematically disadvantaging one group or another would certainly create issues with equity and justice. However, having a system that was perceived to be, or actually is, arbitrary can create a perception, or a reality, of unfairness for the students. Murky or unclear rules, the use of “legacy admissions” (or in this case, selecting those with ties to the program), and the ability for students to manipulate admissions metrics could all add further issues with fairness in the selection process. Finally, the creation of significant delays between medical school graduation and acceptance into a training program would also worsen the selection process by creating detrimental, often undesirable gaps in medical training.

The ability of students to “game” or manipulate the process in any way, particularly those deemed maladaptive or counter to CBHPE, also came to the fore. Using assessment metrics that students can manipulate, such as clerkship or clinical rotation grades, was cited as particularly distressing, as it would incentivize students to hide areas of weakness and discourage them from seeking feedback or help when needed. This is counter to the developmental growth focus of CBHPE. Furthermore, metrics that not all students have an equal chance of attaining were seen as a way to game the selection process while simultaneously presenting potential issues of inequity and unfairness.

Finally, in the worst possible system, many students would turn to artificial intelligence (AI) to craft much of their application. Personal statements and secondary essay prompts would be mostly answered with broad, generalizable text generated by generative AI programs, producing an enormous volume of information for programs to review, often without conveying anything authentic or meaningful about their candidacy. This strategy would lead to the homogenization of the data analyzed in the holistic review process, further complicating the differentiation of students from one another in the selection process. Programs subsequently make arbitrary decisions about who to select after being buried in meaningless data.

### Program Directors’ Perspective of the Worst Selection System or Process

From the perspective of GME program directors, a suboptimal system would center on selecting students they suspected they could not support academically, professionally, or in terms of disability accommodations. This process could also present as programs selecting only candidates they knew would succeed, with little to no intervention, or as a “survival of the fittest” mentality that simply dismissed anyone at any point if they felt they were not “cutting it.” This may also entail recruiting and selecting GME trainees to meet current institutional service needs rather than future societal needs, without any foresight about job prospects in their specialty.

Another aspect of designing the worst possible system would be to ensure it is resource-, time-, and bureaucracy-intensive, yet provides little ability to differentiate among candidates in meaningful ways. This may involve emphasizing information irrelevant to program or student values or program expectations. Application material in this system would not predict who will succeed in the program or take good care of the patients. To review applications, artificial intelligence (AI) would be used to reduce the burden of review, but the AI model would not be adequately trained on the key facets of success in future GME trainees.

Another element of a suboptimal selection system would be to publicly “name and shame” programs for any reason, including publishing how far down their rank list they go to fill their program. It could also result in selection based solely on test scores or the number of hours or publications, creating a “numbers game” when comparing to other programs. Finally, selection systems could create tiers of programs based on “pedigree” or other potentially arbitrary definitions of prestige, resulting in programs not receiving students who would be well aligned with training in CBHPE environments.

### Medical School Leadership/Undergraduate Medical Education Deans’ Perspective of the Worst Selection System or Process

A core feature of the worst possible selection process from a medical school leadership perspective would be obfuscation of metrics or data about students as they apply to GME training programs. This could take the form of outright misinformation about student weaknesses or prior issues, reporting only academic achievement without any discussion of professional behaviors or other domains, or sending only positive attributes while omitting data or evaluations that could be construed as areas for improvement. Such misinformation would be seen as protecting the school’s reputation and ensuring that each student obtains a GME training position, no matter the stakes. This system would not include a meaningful learner handover to future programs and would not provide any information to inform the development of orientations and onboarding processes to support students during the transition.

Another key element in a suboptimal system would be a lack of preparation for the GME position to which the student would be selected. This could take the form of metrics incentivized in medical school (likely at the behest of the GME program) that have little to no relevance to patients or desired outcomes. There would also be a lack of support for struggling students, no additional time available for remediation or learning support, and a lack of focus on student preparation. Growth and development would be discouraged. There would be wide variability in the level of preparation and skill sets of graduates from the same and different schools. There would be no feedback to schools about their graduates and, subsequently, no consequences for schools if they send someone who is entirely unprepared for GME training.

Finally, in the worst possible system, schools could respond to the shadow economy by enforcing it. Allowing the mythology of publication numbers to persist without any intervention to curb this narrative would lead to an explosion of students focusing on research over clinical care. The race to apply to more and more residency programs would spiral further out of control, with medical schools perpetuating the idea that students must apply as broadly as possible to be selected over their peers. Advising would focus entirely on helping students game the selection process rather than on developing their skills and professional identity and aligning them with choosing the right specialty and program for them, ignoring the social accountability mandate altogether.

### Patients/Societal Perspective of a Poor Selection System or Process

The worst possible selection process from a patient perspective would lead to poor patient care and inability to meet community needs. These include selecting students with low empathy or emotional intelligence, and those who prioritize self-interest over patient needs. Rewarding paternalistic approaches to patient care was also identified as poor. Additionally, students who demonstrate that they do not follow through on what they say they will do or seek feedback or help when needed were also a significant concern.

Not meeting the needs of the patient population and/or society was also seen as a key factor in a suboptimal selection process. Having a selection process that does not result in diversity or in physicians who reflect the patient population would be a hallmark of the worst possible system, leading to poorer patient outcomes [15]. Finally, another key element where patient needs would not be met is the design of a selection system that does not ensure that there are enough students to be selected to care for the patients across all necessary specialties and geographic areas, leaving some patients without access to necessary care.

## Discussion

### Comparison of TRIZ Participant Worst System to Current Selection Systems

Components of the worst-possible selection system generated by TRIZ participants bear a familiar resemblance to current selection systems worldwide. This is unsettling. Table 1 summarizes the key elements described during the TRIZ exercise as “the worst selection system” and the corresponding elements identified by the authors as present in current systems. While much of the literature cited is North American-centric, we have endeavored to include examples from the global context when available.

**Table 1:** Perspectives of the key elements that would comprise a faulty selection system as reported by TRIZ participants who acted as proxies on behalf of various interest-holder groups.


KEY INTEREST-HOLDERS IN SELECTION PROCESSES	KEY ELEMENTS IN A WORST POSSIBLE SELECTION SYSTEM DERIVED FROM THE TRIZ EXERCISE	EXAMPLE CURRENTLY IN GME SYSTEMS REPRESENTED IN THE LITERATURE

Students/Applicants	Inequitable or biased selection process (e.g. systematic disadvantaging of a particular group).	Biases against international medical graduates and racial or gender bias in letters of recommendation [16,17,18,19].

	Arbitrary selection process, subject to the whims of the selection program’s leadership who are fallible.	Selection perceived to be arbitrary due to poor data received from applicants and schools leaving program directors to rely on arbitrary scores and uneven group processes [20,21].

	Ability for a student to “game” a system via their assessments.	Students can ensure a higher probability of an “honors” grade by doing a clinical rotation at a specific program over another [22].

	Using metrics where a portion of students cannot attain a good outcome (e.g., score curves and class ranks).	Norm referencing persists to stratify students for comparison in the GME selection process [2].

	Use of artificial intelligence to create personal statements, answer essays, and flood the application with generalizable text data.	Students report using artificial intelligence to generate their personal statements and/or other written application content, while selection committees struggle to discern what is and is not AI-generated [23,24].

	Financial resources can provide unfair advantages (e.g., access to prep courses, ability to interview at more programs).	Students who identify as socioeconomically disadvantaged are less likely to be able to afford test preparation materials and score lower than their peers [25].

GME Training Program Directors	Selecting candidates based on their performance in a common curriculum or approach without accommodations or forgiveness/understanding of their educational progress or journey traveled.	Using score cut-offs to decrease the need to read all applications instead of holistic review for all applications [26].

	Recruiting and selecting students based on service needs within their training setting rather than with any foresight about job prospects within their specialty or community needs.	Selecting GME trainees to provide service but not ensuring they have a job upon graduation in their desired specialty or region. This can also manifest as junior doctors working in generalist specialties for extended periods while repeatedly applying to highly competitive specialties, such as ophthalmology [27,28].

	Resource-, time-, and bureaucracy-intensive process that does little to differentiate candidates (e.g., gathering much information with distracting information that does not predict clinical or other competence).	The current selection process in North America collects a large volume of data but does not meaningfully differentiate among students or reliably predict future success [4].

	Misinformation from the training site about the academic achievements (or lack thereof) and/or progress of a student.	Data in the medical student performance evaluation (a formal document representing the entirety of medical school up to the time of residency application provided by the medical school in the United States) is heterogeneous and may obfuscate student performance [29].

	Inadequate assessment of the true clinical and professional performance of students from their medical school performance evaluation.	Data in medical student performance evaluations are often incomplete, heterogeneous, and opaque, making it difficult for program directors to interpret them adequately [29,30].

	Create a “name-and-shame” culture by equating success in the ranking process with program and PD success.	Programs use post-interview communication as a pressure tactic to convince students to select them and determine where they might be on their rank list; however, this often backfires [31].

	Foster a culture of produce-or-go-unselected within the applicant pool by persistently valuing numbers of publications or hours of participation/leadership.	Program directors and selection committees value the number of publications and extracurriculars over many other factors in selecting students to interview [7,32,33].

	Selecting students who look, think, and act like them and who will ‘fit in’ with the existing group.	Many programs attempt to define and operationalize fit, but it often comes back to how a student “fits” with the current residents and/or faculty [34,35].

Undergraduate Medical Education Leadership	Misrepresent performance of their students to “game” selection rates or pass through “problematic students.”	Many schools have been identified as indiscriminately inflating students’ achievements with adjectives such as “excellent” to help students be selected by programs [36].

	Poorly prepare students to be residents at all, distracting them with non-essential or misincentivized assessments.	The use of assessments for GME selection misincentivizes assessment, leading students to hide areas for growth rather than focus on preparedness for patient care [2].

	Forbidding students from engaging in true apprenticeship and creating a complex observership process and procedural work that precludes the development of clinical acumen and “real-world know-how.”	International medical graduates often encounter significant barriers to observerships in their desired country and specialty, creating additional hurdles to selection [37].

	Allowing the mythology of publication numbers to persist without any intervention to curb the extra- or co-curricular arms race for publications within the medical student population. Allowing continued abuse by faculty within certain fields to capitalize on the “publish or go unmatched” culture makes it challenging to advise students appropriately.	There is a significant shadow economy of effort that capitalizes on students’ publishing to obtain a residency position, particularly in more competitive specialties [7].

	Create policies only based on standardization and conformity.	Professionalism is a competency domain used in selection (or often not to select someone) who may be difficult to train and is a construct that has created significant biases, particularly against students underrepresented in medicine [38].

Patients, Care Partners, Communities & Societal perspectives	Prioritizing the self-interests of the students or programs over those of patients or community needs, whether intentional or not.	Programs consistently (albeit likely unintentionally) prioritize their desired characteristics of student fit over the ability to perform the job they are hiring for or to meet the needs of the patients served by the program [6,39].

	Selecting for a specific group results in a lack of diversity in the selected residents, so that there is little to no representation of the patients/communities they are serving.	Racial concordance between patients and physicians leads to improved communication outcomes, healthcare access by minoritized populations, and lower healthcare expenditures in minoritized populations; however, diversity is often not included or even overtly forbidden as a focus of the selection process [40,41,42,43,44].

	The selection process does not take key patient care and safety measures into account, such as students seeking help when needed or following through on necessary tasks to ensure safe and effective care.	The core tenets of entrustment are important but not a focus of selection as competency measures, and patient needs are often deemphasized in selection systems [39,45].

	There is no consideration of geographic need in the selection process, resulting in physician shortages in rural or less desirable geographic areas.	Rural physician shortages are evident even during training across numerous countries, resulting in access-to-care issues for patients [46,47,48].


First, the current application process for GME training is expensive and time-consuming for students [49]. exams are required to apply, which frequently conveys a high cost. Beyond that, the preparation courses that many student take in order to do well on exams, prepare a successful application, and even to simply send their application to programs are costly. Additionally, much of the testing and application process may introduce bias. The exams themselves have been shown to convey bias in both content and access to preparation courses [50,51]. Preparation courses often expand to cover other elements, such as preparation for interviews, including the multiple mini interview or large-scale situational judgment tests; the ability to pay for these and other preparatory courses or coaching may confer an unfair advantage on a select group of students over others.

Second, bias and the systematic exclusion of certain groups from selection can manifest in other ways. For example, in residency and fellowship selection, these can be overt, such as practices in North America to create additional hurdles for international medical graduates and non-citizens to prevent their selection [52,53]. They can also be more covert, such as the use of “fit” as a selection criterion, which, even when unintended, serves to replicate the current state or status quo of a program and exclude those with new ideas or perspectives, or who do not match the demographics of the program [34,35]. In Europe, for example, there can even be a “pay-to-play” model used to exclude candidates or create gatekeeping by requiring students to complete a PhD or to know/work with a specific person to advocate for them to secure a residency position. Courting the favor of specific individuals may create financial and logistical barriers, most often for those with less exposure to medicine or fewer means to gain access. Additionally, those who have experienced socioeconomic disadvantages are often unable to travel for conferences, global health experiences, or “audition rotations” often necessary to attain a GME position [54].

Even absent bias, there is little evidence that the components of the application, including test scores, grades, experiences, research publications, traditional interview performance, awards, or other elements, translate to future performance or success in relevant outcomes [4,5,55,56,57,58,59,60,61,62,63,64]. Instead, what is often used is what is easy to measure or that looks good for program or university leadership to provide to external interest-holders. It may provide little relevance to whether a student has achieved relevant competencies or will be capable of providing safe and effective patient care [7]. From a future success prediction perspective, test scores often predict future test scores; however, that effect diminishes as students take more clinically relevant exams [5]. The multiple mini interview and situational judgment tests have some predictive value in future clinical performance, particularly around ethical and professional behavior; [62], however, even in programs that can afford to offer these assessments at scale, their discrimination may be limited by the availability of preparation courses to aid students in succeeding at interpreting the questions and answering them “correctly.”

Traditional metrics may also not indicate who will best align with a program’s mission or convey meaningful differences among students [65,66]. Each student is typically highly accomplished, would likely succeed in several programs, and any identified nuance that differs between two students may prove unlikely to be meaningful over time. Programs spend a great deal of time detailing how they can provide all potential pathways a student could want, while students show programs that they can be any version of GME trainee they seek. This may lead to poor alignment between students and programs, dissatisfaction by one or both parties, and, at worst, eventual attrition by the trainee from the GME program. This potential misalignment can be further compounded by a “one-size-fits-all” orientation and support system that provides inadequate accommodations and minimal onboarding at the start of the residency program.

To that end, GME programs may instead focus on a student’s likeability or fit with their program over all else to ensure students are happy in their learning environment and stay in their program [35]. They use some elements of the application, such as letters of recommendation, to interpret their performance in the specialty on rotations but often rely primarily on their interactions with the student to determine whether to rank them highly [67,68]. This may neglect a more rigorous approach that might place greater emphasis on specific competencies as key to selection, or, better, on evidence of a student’s ability to care for patients or meet societal needs, particularly for patients served by the program considering their selection. It also does not address the biases that the selection committee may have, individually or collectively.

Finally, most, if not all, interest-holders in current selection processes have an incentive to deceive or turn a blind eye to concerns, depending on their positionality in the process. Medical schools may benefit from obfuscating information conveyed to residency training programs to ensure that students are selected for residency programs. When training programs realize that information is missing, they mistrust the schools and the information schools send as part of the application process [69]. Students may also engage in deception by hiding deficiencies as part of the process to ensure they are chosen by their desired program [2]. All of this gamesmanship may result in a high degree of mistrust among all parties as they respond to external pressures and incentives rather than optimizing the selection process for all involved. Meaningful learner handover (by faculty, program, or student) for the purposes of support and to ensure success and thriving is not prioritized and is often discouraged [70,71]. This process undermines CBHPE’s drive for a growth mindset, feedback-seeking behavior, and student-directed learning.

### Recommendations to Improve Selection Processes to Align with the Goals of CBHPE

Based on our TRIZ exercise and a comparison of elements of the “worst” selection system with those of the current system, we offer several recommendations to improve selection processes and align them with the goals of CBHPE. These recommendations are derived from the TRIZ exercise and expanded by the authors here to situate them within the existing literature and provide a synthetic vision for a way forward. We also provide a prioritization of the solutions as recommended by the authors in Appendix 1.

#### Considerations Related to the Core Components of CBHPE

There are a number of potential solutions aligned with the core components of CBHPE, many of which are mirrored in the report provided by the Coalition for Physician Accountability’s UME-GME Review Committee (UGRC) in calling for the implementation of the core components of CBHPE to address many of the issues within the transition from UME to GME [72,73]. Selection processes need to be socially accountable while also meeting student and program needs. The selection process should be redesigned to prioritize aligning candidates with patient and societal needs, a foundational goal of CBHPE [2,39]. By leveraging competency frameworks that focus on socially accountable outcomes, medical schools can provide students’ competency data to inform the selection process [74]. This focus would allow schools to highlight specific clinical competence, skill sets, and domains of excellence relevant to patient care while simultaneously ensuring students are prepared for the patient care demands expected of them on day one of GME training [75].

Competency frameworks, such as the Core EPAs for Entering Residency, would provide transparent expectations for students during the assessment process, helping them focus their energy [76,77,78,79]. This can replace funneling efforts into the shadow economy of research publications and other potentially irrelevant check-boxing activities that detract from preparedness for patient care and the social accountability mandate. By engaging all interest-holders in ensuring longitudinal buy-in and clearly articulating what to focus on – including clinical reasoning, interprofessional teamwork, patient communication, or other domains core to patient care – competency frameworks can align student expectations with both patient/societal needs and preparedness for residency. Leveraging the selection process to incentivize the same does so by bringing these domains together into a cohesive whole focused on both patient care and student readiness.

A selection process that truly incentivizes growth mindset in students and a transparent, honest handover of growth-oriented data from medical schools to GME training programs would create an ideal transition for students from one phase of training to the next [80]. The clear communication of student attainment of competence across a variety of domains would allow programs to both better understand how to support entering GME trainees as well as ensure they are selecting students they actually are capable of supporting based on their available resources. By developing a deep understanding of any gaps each entering trainee has, GME programs may provide necessary onboarding, training, schedule adjustments, or support structures, including accommodations, to ensure GME trainee success in their new training environment and to optimize the delivery of safe and effective patient care by early GME trainees. Additionally, normalizing that each GME trainee has gaps and encouraging growth, development, feedback, and help-seeking provides a safe learning environment and supports GME trainees in providing excellent patient care.

#### Considerations Related to Diversity and Equity

Diversity is essential to ensure optimal patient care. As such, it is imperative to prioritize selecting a diverse cohort of students who represent the patients/communities served. Explicitly prioritizing diversity in all its forms should be included in the holistic review of applications and the mission-aligned selection of students for each program, with an eye toward the patient population served. Pathway programs must also be prioritized as early as grade school to optimize the diversity of those entering the profession and create a workforce that truly aligns with the patient population served [81].

Equity within the assessment process in medical schools as well as the application review and ultimately the selection process itself is also a core feature of improving selection [82]. Students must be treated equitably, without one group being advantaged over another, to achieve a more ideal selection process. Leveraging criterion-referenced measures aligned with competency frameworks, implementing programmatic assessment principles, and focusing on a truly holistic review are key steps toward achieving equity in selection and in the assessments that feed into this process [83]. Implicit bias training may also play a significant role in both the assessments that drive selection and the selection processes themselves, and it should be mandated for all involved in both selection and assessment [84].

#### Logistical and Structural Considerations

When students do not meet competency standards or are not selected, there must be processes in place to support them. Those who repeatedly prove they are unable to meet the necessary thresholds either in patient care domains or professional behaviors should be given options within the dismissal process, sometimes referred to as “compassionate offramps” [85,86]. Advanced degrees for time spent in medical school and advising on potential alternatives are essential support structures for those who should not graduate from medical school and advance to GME training. For those who are not selected for their desired specialty, there must be pathways for them to continue their growth and development. Clinical placements in clinical or geographic areas of need could aid continued clinical learning and skill development while also supporting further growth in competency domains relevant to future selection, either within the same or an alternative chosen discipline.

Finally, numerous essential structural elements can improve the selection process. Ensuring adequate time for a holistic review of each application, with an eye toward adequate support and mission alignment with the program, is key. To that end, technological elements of the selection process, including artificial intelligence to augment the holistic review, could be beneficial, so long as they are trained and monitored for bias [87]. Innovations such as preference signaling for students to show genuine interest in programs have already created better alignment in many instances but would benefit from greater transparency between programs and students regarding their use [88]. If these elements prove insufficient to facilitate transparency, mitigate bias, and better incentivize competence and social accountability, a lottery may be necessary to level the selection process to facilitate the adoption of competency-based education and better ensure preparedness for GME training and the provision of patient care instead of students focusing on checking boxes within the shadow economy for residency selection [89,90].

## Limitations

We performed TRIZ with the authors acting as, or “wearing the hat” of, the various interest-holder groups. The small sample size of eight participants and a single 60-minute TRIZ exercise, while exhaustive of the ideas of those present, does not fully represent the breadth of what could have been generated with iterative sessions and varied groups of participants.

Additionally, while all the authors have varied experience with selection and CBHPE and some have significant depth of expertise in one or both, they do not represent each of the interest-holder groups included. This could create biases toward or away from, or misrepresentations of, these various groups that may have skewed our results, as the data does not come directly from each of these individual interest-holders’ perspectives. The potential influence of researcher framing and interpretation of both the exercise itself and the subsequent analysis must be taken into account when interpreting the results and the recommendations advanced by the authors.

In order to keep the TRIZ exercise manageable and focused, we chose only to sample around selection in medicine and particularly selection to GME training. The exclusion of other health professions may limit the transferability or generalizability of these findings beyond medicine; however, a similar exercise or approach is likely to be beneficial in other contexts.

Additionally, while multiple countries and specialties were represented, there were no attendees or authors from several contexts that could benefit from representation in this discussion, as selection and CBHPE adoption and adaptation around the globe face unique contextual challenges that must be further explored to be adequately addressed. For example, many of the authors are familiar with the traditional undergraduate-to-graduate medical education flow, whether into general practice or specialty training right after undergraduate training. However, in several contexts, undergraduate trainees move directly into practice, thus making the undergraduate selection process more high-stakes and creating additional issues not explored here.

## Next Steps

To further address the limitations presented and expand upon these recommendations, next steps would entail engaging a broader swath of stakeholders, including students, patients, and societal representatives. Additionally, engaging with decision-makers at national organizations to implement the changes highlighted above would be necessary to continue moving the needle forward in addressing the wicked problem at the intersection of selection and CBHPE.

## Conclusion

The TRIZ exercise provided the authors with the opportunity to be creative and innovate at the intersection of CBHPE and selection, allowing them to see this wicked problem from a new vantage point. This process illuminated the existing pathology and provided a forum for deliberate co-creation and positive change.

Several elements of the worst possible selection system exist in our current systems presenting barriers for a way forward in aligning selection and CBHPE. We present several potential solutions to move this discussion forward toward action. There is a clear need for social accountability to be embedded in all areas of health professions education, including CBHPE [91] and selection. This will ensure that all interest holders, including students, programs, medical schools, and most importantly patients and communities, can thrive.

## Disclaimer

The views expressed herein are those of the authors and not necessarily those of the American Medical Association, or other Federal or Governmental Agencies or other organizations with whom the authors are affiliated.

## Additional File

The additional file for this article can be found as follows:

10.5334/pme.2596.s1 Appendix 1. Complete results of TRIZ exercise.
